# The Effectiveness of a Pharmaceutical Care Model on Adherence to Antiretroviral Therapy: A SAME-Based Cohort Study in Brazil

**DOI:** 10.15171/apb.2017.056

**Published:** 2017-09-25

**Authors:** Marli Gerenutti, Adriana Michel Vieira Martinez, Cristiane de Cassia Bergamaschi

**Affiliations:** ^1^Department of Pharmaceutical Sciences, Universidade de Sorocaba (UNISO), Sorocaba, Brazil.; ^2^Specialized Municipal Assistance Service (SAME) in HIV/AIDS, Sorocaba, Brazil.

**Keywords:** Adherence rates, Antiretroviral agents, Maintenance of antiretroviral therapy, Medication adherence, Pharmaceutical care

## Abstract

***Purpose:*** To verify the effectiveness of a pharmaceutical care model developed by the Specialized Municipal Assistance Service in Sorocaba, Brazil, on adherence to ART among patients infected with HIV.

***Methods:*** A cohort study compared adherence to ART in two groups of patients: intervention group (patients assisted with pharmaceutical care, n=130) and non-intervention group (patients attended by the habitual dispensing process, n=229). Antiretroviral adherence was measured by the number of pharmacy refill records in a six-month period. The relationship between the use of other drugs for the treatment of opportunistic infections and the adherence rate in the intervention group and the correlation between adherence and viral load and CD4 lymphocytes were also assessed.

***Results:*** Higher adherence rates were observed in the intervention group (p<0.05). The use of others drugs did not influence adherence to ART (p=0.30). There was a positive correlation between adherence and the percentage of patients in the intervention group with undetectable viral loads (p=0.0004) and higher levels of CD4 lymphocytes (p=0.0024).

***Conclusion:*** The pharmaceutical care model developed by the SAME improved patient adherence to ART as well as clinical outcomes.

## Introduction

Adherence to antiretroviral therapy (ART) is fundamental to the success of HIV treatment because low adherence diminishes the effectiveness of the drugs, worsens clinical outcomes in patients, and contributes to the dissemination of resistant HIV strains.^1^ Pharmacist interventions in patients infected with HIV can contribute to the maintenance of antiretroviral therapy and improvement in clinical outcomes.^2^ Partial adherence or non-adherence to ART is one of the main barriers to its effectiveness.^3,4^ In this context, studies that identify the benefits of different pharmaceutical care practices in supporting adherence to antiretroviral therapy are necessary.

In 1996, Brazil was the first country to guarantee to HIV/AIDS patients the right to receive all of the drugs needed for their treatment free of charge through the public system.^5^ A pharmaceutical care model specializing in HIV/AIDS was developed by the Specialized Municipal Assistance Service (SAME) in Sorocaba City, SP, Brazil. This model consists of a pharmaceutical consultation that seeks information regarding medications and provides explanations to patients about their treatment, enhancing co-responsibility and contributing to adherence to therapy. To date, no study has been conducted in the SAME to verify the clinical benefit of these models. The present study aimed to describe the effectiveness of this model of pharmaceutical care in supporting adherence to antiretroviral therapy. Also, the relationship between adherence and viral load and CD4 lymphocyte values was evaluated.

## Materials and Methods

### Study Design and local of Study

A cohort study that followed the patients over a period of six-months. The study was developed at the SAME, in Sorocaba, State of São Paulo, Brazil. The service has a multi-disciplinary team.

### Study population 

The study population consisted of all patients infected with HIV and taking antiretroviral therapy enrolled in the pharmacy SICLOM (Digitized System of Drug Logistic Control) during the period of study. Patients aged over 12 years infected with HIV and receiving antiretroviral therapy for at least one year and registered at SICLOM were included. Patients, who transferred to other services, received antiretroviral therapy for a short interval, were pregnant, or died during the study period, were excluded.

### Allocation

The patients were divided into two groups: pharmaceutical intervention (patients seen under the model of pharmaceutical care developed at the SAME by the responsible pharmacist group) and non-intervention group (patients that refusal to participate in the intervention group or was an incompatibility in appointment time times).

### The pharmaceutical care model 

This model consisted of one pharmaceutical consultation with patients or parent or guardian of minor patients. During the consultation, a chart was prepared for each patient with information regarding their medical prescription and their antiretroviral treatment-taking habits. The table contained information that oriented patients to the appropriate times to ingest their drugs, the number of pills to take, and images of the antiretrovirals. Possible interactions between drugs and food were explained verbally and separated in the schedules at different times. The pharmacist explained the main adverse effects that occur with the use of antiretroviral therapy and how to manage them. At the consultation, self-adhesive tags with standardized drawings were placed in vials and blisters of drugs with the intent of reinforcing the information given to the patient as far as time, dosage and interval of drug usage.

### Adherence to antiretroviral therapy

Adherence to antiretroviral therapy was verified by the number of pharmacy refill records^6^ in a six-month period. Patients who received all six refills (100%) were classified as adherent. Those that received five monthly refills (80.0%) were classified into the minimum effective adherence. Patients who received between one and four refills (below 66.7%) were classified into the low adherence category. Finally, patients who did not refill their medications during the study were classified as non-adherents.^7^ The association between adherence to antiretroviral therapy and the prescription of drugs for the treatment of opportunistic infections was verified in the intervention group only since SICLOM does not permit access to this information.

### Correlation between adherence to antiretroviral therapy and viral load and CD4 lymphocyte values

Viral load and CD4 lymphocyte values were collected from the clinical charts of patients in the intervention group at close to the day of pharmaceutical consultation and six months after consultation. It was not possible to obtain the viral load values in the non-intervention group and some of the patients in the intervention group due to the absence of this information in their clinical charts.

Patients were classified into: patients with an undetectable viral load (less than 40 copies/ml), patients with at least a 90% reduction in the viral burden (about to the initial measurement), and patients with an unsatisfactory response to ART. The correlation between adherence to ART and viral load levels six months after the initial pharmaceutical consultation was measured based on the above data.

The CD4 cell count was considered in this study because it is a classic marker of disease progression, with increased measurement challenges in advanced phases of infection. The values were considered to be altered when the results were below 500 cells/mm³ in proportion to the total number of lymphocytes.^8,9^ The correlation between adherence to ART and increase in CD4 levels (>500 cells/mm³) was measured six months after the initial pharmaceutical consultation.

### Statistical Analysis

Chi-square tests were used to evaluate levels of adherence among the groups. The correlation between adherence and the levels of viral load and CD4 lymphocytes was established by the Spearman correlation coefficient. Statistical analyses were performed using Bioestat software 5.3, Institute of Sustainable Development Mamirauá, p=5%.

## Results and Discussion

The sample consisted of 359 patients, with 130 in the intervention group and 229 in the non-intervention group. The majority of patients were adults. Approximately 50.7% of patients were male, and 49.3% were female. It was observed that the pharmaceutical care model developed in the SAME was associated with higher rates of adherence to ART and decreased the rates of treatment abandonment.

Table 1 compares the frequency of pharmacy refills of antiretrovirals between the groups. In the non-intervention group, it was observed that 81 patients (35.3%) adhered to the medications. Forty-nine and 24 patients in the intervention group had ideal adherence and minimum adherence to antiretrovirals. These results indicate that the intervention of pharmaceutical consultation improved adherence to ART and reduced the abandonment rate compared to the non-intervention group (p=0.001).

**Table 1:** Absolute and relative frequency to adherence to antiretroviral in patients of the intervention and non-intervention groups (n=359).

**Groups**	**Adherence**	**Minimum effective adherence**	**Low adherence**	**Non-adherent**
**NIG**	57 (24.9%)	24 (10.4%)	56 (24.5%)	92 (40.2%)
**IG**	49(37.7%)*	24 (18.4%)*	48 (36.1%)	10 (7.7%)*

NIG: non-intervention group; IG: intervention group; Chi-square, * p<0.001.

The results of a systematic review by Saberi et al^6^ showed that the most common methods of adherence assessment were based on medication refill records, followed by patient self-report and electronic drug monitoring using medication event monitoring systems. The Brazilian studies that utilized SICLOM to gather data about antiretroviral adherence considered the system as an important tool for monitoring irregular adherence patterns or risk of treatment abandonment.^10^

Court et al^11^ evaluated patient adherence to antiretrovirals using the same methods employed in this study. The study verified the association between adherence, as assessed by pharmacy refill records, and virologic failure to develop a clinical tool for use in antiretroviral programs in low-middle income settings. They observed that the risk of virologic failure fell by 73% with each 10% increase in adherence measured using pharmacy refill records over a four-month period.

The present study demonstrated that the intervention of a pharmaceutical consultation aided the monitoring of patients by SICLOM reduced treatment abandonment rates. Hirsch et al^12^ also verified improved adherence to antiretroviral therapy among patients accompanied by the pharmacy service in the Department of Health Services of California. These patients were more likely to remain on one type of drug regimen throughout the year and had a fewer change in regimens.

Table 2 shows the levels of antiretroviral adherence associated with opportunistic infection treatment among the intervention group. The non-intervention group is not represented in the table due to the lack of information on the usage of concurrent drugs in SICLOM. However, in the intervention group, 30 out of 130 patients evaluated received both antiretroviral and opportunistic infection treatment, with no statistically significant differences observed on to adherence and opportunistic infection treatment (p=0.3591).

**Table 2:** Absolute and relative frequency to adherence in IG patients to antiretroviral and antiretroviral therapy associated with opportunist infection therapy (n=130).

**Treatments**	**Adherence**	**Minimum effective adherence**	**Low adherence**	**Nonadherent**	**Total**
IG (ART)	34 (34.0%)	21 (21.0%)	37 (37.0%)	8 (8.0%)	100 (100%)
IG (ART+OIT)	15 (50.0%)	3 (10.0%)	10 (33.3%)	2 (6.7%)	30 (100%)
Total	49 (37.7%)	24 (18.4%)	47 (36.1%)	10 (7.7%)	130 (100%)
IG: Intervention group; ART: Antiretroviral therapy; OIT: Opportunist infection therapy; Chi-square, p=0.3591.					

In the intervention group, a positive and significant correlation coefficient was observed between treatment adherence and undetectable viral load values and increased CD4 lymphocyte levels. Patients with maximum levels of treatment adherence showed the highest levels of undetectable viral load at 86.0% (Table 3).

**Table 3:** Correlation between adherence from patients of the intervention group and the undetectable viral load values and increase in CD4 lymphocyte levels (n=130)

**Number of patients (%)**	**Adherence**	**Minimum effective adherence**	**Low adherence**	**Non-adherent**
Undetectable viral load*	49 (86.0%)	18(72.0%)	24 (58.5%)	0 (0)
Increase in CD4 levels**	46 (80.7%)	18 (72.0%)	20 (48.8%)	2 (28.6%)
Total	57 (100%)	25 (100%)	41 (100%)	7 (100%)

Spearman correlation coefficient (* r=0.95, p=0.0004) (** r=0.97, p=0.0024)

Ma et al^13^ observed results consistent with the findings of our study. The study evaluated the efficacy of a clinical pharmacist intervention and verified the decrease or maximum suppression of the viral load and the increase in CD4 lymphocytes. Patients who had less than 70% adherence to antiretroviral treatment showed a five-fold increase in disease progression risk. Comparatively, patients who showed greater than 90% adherence had an increased length of time to viral failure, increased CD4 lymphocyte counts, and less disease progression risk and posterior death.^14^ However, there is conflicting evidence on defining the minimum threshold of adherence for the effectiveness of antiretroviral therapy. HIV care providers continue to aim to achieve adherence levels of 95% or more, although the clinical significance to the patient of maintaining a particular adherence percentage remains unclear.^15^

Although our results demonstrate the benefits of a pharmaceutical care model implemented by the SAME, the rate of patients with the ideal adherence can be improved. This model is the main tool used by the clinical pharmacist at the SAME and is in the process of further improvement. We noted that teenagers, who composed only approximately 5% of participants of the study, had increased doubts regarding treatment, suggesting that the model requires adaptation to the needs of different subpopulations, such as modification in drug therapy, among others.

One disadvantage of the pharmaceutical care model was the inability to verify if the drugs were correctly administered. Even vials with sensors for the electronic monitoring of medication-taking can be altered. However, information regarding the most recent refill records made it possible to estimate the adherence of patients to ART, and if needed, reinforcement of medication-taking could be provided during the refill encounter. This study described the pharmaceutical service of the SAME and demonstrated the benefits of the model for adherence to therapy and response to certain clinical parameters. This tool could help identify patients who require intervention to improve the adherence.

## Conclusion

The pharmaceutical care model developed at the SAME positively influenced patient’s adherence to antiretroviral therapy as well as reduced viral loads and increased CD4 lymphocytes levels.

## Acknowledgments

The authors would like to acknowledge the Specialized Municipal Assistance Service (SAME) in HIV/AIDS, Sorocaba, Brazil.

## Ethical Issues

The study was approved by the Committee on Ethics in Research on Human Beings of the University of Sorocaba (number. 18/2011).

## Conflict of Interest

The authors declare no conflict of interests.
